# Discussing the Score of Cardioembolic Ischemic Stroke in Chagas Disease

**DOI:** 10.3390/tropicalmed5020082

**Published:** 2020-05-26

**Authors:** Fernanda de Souza Nogueira Sardinha Mendes, Mauro Felippe Felix Mediano, Rudson Santos Silva, Sergio Salles Xavier, Pedro Emmanuel Alvarenga Americano do Brasil, Roberto Magalhães Saraiva, Alejandro Marcel Hasslocher-Moreno, Andrea Silvestre de Sousa

**Affiliations:** Instituto Nacional de Infectologia Evandro Chagas, Oswaldo Cruz Foundation (Fiocruz), Avenida Brasil 4365, Rio de Janeiro 21045-900, Brazil; mauro.mediano@ini.fiocruz.br (M.F.F.M.); rudson.santos@ini.fiocruz.br (R.S.S.); sergio.xavier@ini.fiocruz.br (S.S.X.); pedro.brasil@ini.fiocruz.br (P.E.A.A.d.B.); roberto.saraiva@ini.fiocruz.br (R.M.S.); alejandro.hasslocher@ini.fiocruz.br (A.M.H.-M.); andrea.silvestre@ini.fiocruz.br (A.S.d.S.)

**Keywords:** Chagas disease, stroke, risk assessment

## Abstract

Chagas disease is an important infection in Latin America but it is also reported in non-endemic countries all over the world. Around 30% of infected patients develop chronic Chagas cardiopathy, which is responsible for most poor outcomes, mainly heart failure, arrhythmias and thromboembolic events. Of all thromboembolic events, stroke is the most feared, due to the high probability of evolution to death or disability. Despite its importance, the actual incidence of cardioembolic ischemic stroke in Chagas disease is not completely known. The Instituto de Pesquisa Evandro Chagas/Fundação Oswaldo Cruz (IPEC-FIOCRUZ) score aims to propose prophylaxis strategies against cardioembolic ischemic stroke in Chagas disease based on clinical risk–benefit. To date, the IPEC-FIOCRUZ score is considered the best tool to identify patients for stroke prophylaxis in Chagas disease according the Latin American guideline and Brazilian consensus. It can prevent many cardioembolic strokes that would not be predicted, by applying the current recommendations to other cardiopathies. However, the IPEC-FIOCRUZ score still requires external validation to be used in different Chagas disease populations with an appropriate study design.

## 1. Chagas Disease and Stroke

Chagas disease (CD) is an important protozoan infection in Latin America where it remains one of the most important public health problems due to the high morbidity and mortality associated with the chronic cardiac form of the disease [1]. Nowadays, CD cases are also being reported worldwide due to international migration and autochthonous cases outside Latin America that may occur due to organ transplantation, blood transfusion and congenital transmission. The World Health Organization estimates that around six million people are infected by *Trypanosoma cruzi* [2]. Around 30% of infected patients develop the chronic cardiac form of the disease, responsible for most of the poor outcomes, mainly heart failure, arrhythmias and thromboembolic events [3,4]. Thromboembolic events in Chagas heart disease are more frequent than in other cardiopathies, even with similar degrees of systolic dysfunction [5], inferring that chagasic cardiopathy has a higher emboligenic potential. For instance, a much higher frequency of chagasic stroke in patients without vascular risk factors is observed when compared to the non-Chagas cohort [6]. According to De Paiva Bezerra et al., in a 32-patient cohort, it was found that 87.5% had cardioembolic stroke, 9.4% had large intracranial artery atherosclerotic stroke and 3.1% had an undetermined cause [7]. Of all thromboembolic events, stroke is the most feared, due to the high probability of death or disability. Additionally, chagasic cardiopathy has unique characteristics such as apical aneurism, an important risk factor for cardioembolic events [6], which can limit the efficacy of prophylaxis in cardioembolic ischemic stroke (CIS). Thus, it is important to identify the main factors associated with an increased risk of cardioembolic ischemic stroke in CD and their mechanisms in order to propose tailored strategies to prevent it.

One study estimated that the annual incidence of CIS in a cohort of patients with CD was 3% in all subjects, and 4.4% in the high-risk subgroup, with a mean follow-up period of 5.5 years [8]. In a large Brazilian study [9], 24.4% of the stroke patients had positive serology for CD. A Colombian case-control study found similar results, detecting that *T. cruzi* infection was more frequent and statistically significant in stroke cases (24.4%) than controls (1.9%) [10]. Moreover, CD stroke patients had lower rates of cardiovascular risk factors but higher rates of multiple cerebrovascular events than non-Chagas patients did. In Colombia [11], CD was much more frequent among patients with stroke than in those with other diseases (OR = 12.13; 95% CI, 3.64 to 71.4). The cumulative incidence of death due to stroke among patients with CD was described to be 4.8% in a 10-year follow-up study [10], but new studies are needed to establish the mortality associated with stroke due to CD. Therefore, it is fundamental to identify strategies to prevent these events in patients with CD.

## 2. The IPEC-FIOCRUZ Stroke Score for Patients with Chagas Disease

The Instituto de Pesquisa Evandro Chagas/Fundação Oswaldo Cruz (IPEC-FIOCRUZ) score was developed, proposing prevention strategies for CIS in CD based on a risk–benefit analysis [9]. In the validation study, consecutive patients with positive serology for CD were followed for at least one year. The investigators studied two specific treatments: oral anticoagulation drugs and acetylsalicylic acid. The events were all cases of ischemic stroke or transient ischemic attack, defined as the cardioembolic type according to the Trial of Org 10172 in Acute Stroke Treatment (TOAST) classification [12]. Currently, the only group with a well-established proposal of prophylaxis, despite the CD diagnosis, is atrial fibrillation. The IPEC-FIOCRUZ score was built to fulfill the lack of knowledge of the individual risk–benefit of prophylaxis for cardioembolic stroke in the Chagas cardiopathy population not covered by the available scores. In this way, patients with previous ischemic stroke, intracavitary thrombus or atrial fibrillation were not included in this analysis because they already had a prophylaxis indication according to the in-force recommendations [13]. In this setting, fifty-two patients were observed, to evaluate the hemorrhagic risk of anticoagulation.

Four items were relevant in the IPEC-FIOCRUZ score: age >48 years, primary change of ventricular repolarization, systolic dysfunction and apical aneurysm of the left ventricle. The score ranges from zero to five points, and the group suggested primary prophylaxis with anticoagulants for those scoring ≥4 [6]. A total of 7.3% of the cohort was in a high-risk group (4–5 points) and 45% of them had the CIS diagnostic. This score was included in the Brazilian consensus and Latin American guideline for treatment of CD as an important strategy for detection of patients at increased risk of CIS [3,8,14].

Recently, Montanaro et al. [14] conducted a retrospective study that aimed to evaluate the performance of the IPEC-FIOCRUZ score in identifying patients at high risk of CIS. The authors included patients admitted to the SARAH Hospital of Rehabilitation from 2009 to 2013, diagnosed with ischemic stroke and CD. The mean time from ictus to inclusion was three years, and the mean follow-up time in the rehabilitation program was five years. By the TOAST etiological classification, 45% of the events were cardioembolic, 8.2% were atherothrombotic and in 45% the etiology of the event was not determined. The authors calculated the current IPEC-FIOCRUZ score of the patients and showed that 69.6% of the stroke patients had an IPEC-FIOCRUZ score of 1 or 2, and only 30.4% had a score of 3 or more. In addition, from those with cardioembolic etiology, 55% of patients were classified as low risk (IPEC-FIOCRUZ score <3). The authors concluded that more than half of the patients with cardioembolic strokes were misclassified as low risk and suggested that the current guidelines for stroke prevention in CD should be reviewed. However, there are several issues regarding population type, study design and data analysis that limited the authors’ conclusions. The IPEC-FIOCRUZ score was built to predict individual cardioembolic risk and the benefit of each available treatment (i.e., warfarin and acetylsalicylic acid); however, Montanaro et al. [14] used this score in a population with known ischemic stroke from all etiologies (not only cardioembolic cases), in a retrospective analysis to check if the patients had a high score. As 55% of the cardioembolic events occurred in patients in the low-risk strata, Montanaro et al. supposed that if the score would have been applied before the occurrence of vascular ictus, the number of patients at risk would have been underestimated. However, the rationale used by the authors may be misleading, as discussed below. There were also no data regarding the previous use of anticoagulant or antiplatelet therapy by the patients who suffered stroke.

The purpose of a risk assessment tool (i.e., risk score) is to assess individual risk and propose recommendations of actions according to the estimated risk. In most cases, the group with the highest individual risk is much smaller than the low-risk group, which results in a higher absolute number of outcomes in the low-risk group. This apparent paradox has been described for many years in relation to sudden death [15,16]. Although the patients at high relative risk for sudden cardiac death can be identified by their risk factors, the greatest number of sudden death cases occurs in patients not previously determined to be at high risk. This paradox makes it difficult to adopt preventive measures on a large scale [17]. Using the example of Geoffrey Rose in his paper [18], when we think of the occurrence of Down’s syndrome births in regards to maternal age, younger mothers are at minimal risk; but because they are the majority, they generate half the cases. The lesson is that “a large number of people with small risk may give rise to more cases of disease than the small number who are at high risk”. If we think with this approach in cardioembolic stroke in CD, most of the cases are in the low-risk population. This is one of the reasons why it is not right to validate the risk score by applying it to the prevalent cases. To validate a score, we need to apply it to the whole population that is at risk of the event—in this case, people with CD free of cardioembolic stroke—and compare the predicted events with the observed ones. This will enable the estimation of discrimination and calibration measures.

The second important issue: Is it appropriate to include all patients with cardioembolic stroke, even those with previous stroke and atrial fibrillation, and apply the IPEC-FIOCRUZ score? These predictors were not included in the score because they already had an indication for stroke prophylaxis before the risk assessment. From a practical point of view, it does not make sense to use a score in helping to decide whether to prescribe anticoagulants when the patient should already be taking this therapy [19].

## 3. Perspectives for Scoring Stroke Risk in Chagas Disease

The IPEC-FIOCRUZ score was validated in a single center with a relatively small sample size. Therefore, it requires external validation to be used in different populations with CD, but an appropriate study design is necessary. The area under the Receiver Operating Characteristic (ROC) curve of this model was 0.90 (95% CI 0.86 to 0.94), which is the best parameter to evaluate score accuracy until an external validation in a different cohort is done. There are increasingly acceptable standards in the literature regarding development, validation and update of prediction/decision models [20]. A study to validate the IPEC-FIOCRUZ stroke score should include subjects with CD, initially stroke-free, but at potential risk of stroke occurrence in the future. Additionally, the subjects should represent the population where a decision to introduce preventive therapy is required. A prediction score in this case should be applied when observation starts, where potential courses of actions are in discussion, and not for those that should be already taking prophylactic therapy, i.e., for previous cardioembolic stroke, intracavitary thrombus or atrial fibrillation. Later, the discrimination ability and calibration would be estimated when comparing the predictions with the observed events.

The validation and update of useful prediction/decision models are always advisable, mainly where the model may be applicable in several different settings (e.g., rural cohorts, non-endemic countries, different health units, etc.). However, this effort should follow standards currently acceptable for an appropriate interpretation. These appropriate validation studies are welcome and expected for all models of a devastating neglected disease.

## 4. Conclusions

Until now, the IPEC-FIOCRUZ score remains the best stroke prophylaxis tool in CD according to the Latin American guideline and Brazilian consensus [3,8], which is capable of identifying patients at risk and avoiding cardioembolic strokes that would not be avoided by applying the usual recommendations for stroke prophylaxis in other cardiopathies.
